# An Ultrasound Model to Predict the Short-Term Effects of Endovascular Stent Placement in the Treatment of Carotid Artery Stenosis

**DOI:** 10.3389/fcvm.2020.607367

**Published:** 2021-01-22

**Authors:** Sheng-Jiang Chen, Rui-Rui Liu, Yi-Ran Shang, Yu-Juan Xie, Xiao-Han Guo, Meng-Jiao Huang, Xiao-Feng Yang, Qi-Zhi Fu, Ji-Sheng Qi, Dong-Yan Shen, Jia-Yan Li

**Affiliations:** ^1^Department of Ultrasound, The First Affiliated Hospital, College of Clinical Medicine of Henan University of Science and Technology, Luoyang, China; ^2^Department of Neurology, The First Affiliated Hospital, College of Clinical Medicine of Henan University of Science and Technology, Luoyang, China; ^3^Department of Vascular Surgery, The First Affiliated Hospital, College of Clinical Medicine of Henan University of Science and Technology, Luoyang, China; ^4^Department of Ultrasound, Luoyang Dongfang Hospital, The Third Affiliated Hospital of Henan University of Science and Technology, Luoyang, China

**Keywords:** carotid artery, neurological deficit score, stent placement, ultrasound, model

## Abstract

**Purpose:** The present study aimed to explore the predictive ability of an ultrasound linear regression equation in patients undergoing endovascular stent placement (ESP) to treat carotid artery stenosis-induced ischemic stroke.

**Methods:** Pearson's correlation coefficient of actual improvement rate (IR) and 10 preoperative ultrasound indices in the carotid arteries of 64 patients who underwent ESP were retrospectively analyzed. A predictive ultrasound model for the fitted IR after ESP was established.

**Results:** Of the 10 preoperative ultrasound indices, peak systolic velocity (PSV) at stenosis was strongly correlated with postoperative actual IR (*r* = 0.622; *P* < 0.01). The unstable plaque index (UPI; *r* = 0.447), peak eccentricity ratio (*r* = 0.431), and plaque stiffness index (β; *r* = 0.512) moderately correlated with actual IR (*P* < 0.01). Furthermore, the resistance index (*r* = 0.325) and the dilation coefficient (*r* = 0.311) weakly correlated with actual IR (*P* < 0.05). There was no significant correlation between actual IR and the number of unstable plaques, area narrowing, pulsatility index, and compliance coefficient. In combination, morphological, hemodynamic, and physiological ultrasound indices can predict 62.39% of neurological deficits after ESP: fitted IR = 0.9816 – 0.1293β + 0.0504UPI – 0.1137PSV.

**Conclusion:** Certain carotid ultrasound indices correlate with ESP outcomes. The multi-index predictive model can be used to evaluate the effects of ESP before surgery.

## Introduction

The carotid arteries are important blood vessels that supply blood to the brain and head. Carotid artery stenosis is an important risk factor for ischemic stroke. Early and proper intervention of carotid artery stenosis can help to prevent ischemic stroke (1–3).

In recent years, with the application of cerebral protection devices, endovascular stent placement (ESP) has become widely popularized among neurosurgeons in the treatment of patients. However, inappropriate selection of indications, variable postoperative outcomes, and in-stent restenosis are still common problems (4–6).

The present study analyzed the correlation between the neurological status of patients 30 days after surgery with ESP and examined ultrasound examination indices before surgery in 64 patients with carotid artery stenosis treated with ESP. Furthermore, a preoperative ultrasound prediction model was established to explore the ability of ultrasound to predict the neurological status of patients following ESP to provide an objective basis for the development of a reasonable clinical treatment regime.

## Information and Methods

### Subjects

From January 2008 to September 2016, a total of 64 patients (male, *n* = 35; female, *n* = 29) with carotid artery stenosis-induced acute ischemic stroke, who were treated by ESP at hospitals affiliated to our study center. And who were matched in blood pressure, blood glucose, body mass index and other basic data, were enrolled. Patients whose body mass index, blood pressure, fasting blood-glucose, serum low density lipoprotein, fibrinogen, CRP and other indicators beyond 20% of the normal threshold were excluded from the study cohort. The age range of patients was 43–79 years, with an average age of 65.1 ± 8.2 years. The interval from diagnosis of carotid artery stenosis to treatment ranged from 1 to 45 months, with an average interval of 22.4 ± 6.5 months.

Patients presented with the following stroke-related symptoms: vertigo (*n* = 31), tinnitus (*n* = 8), headache (*n* = 9), blurred vision (*n* = 11), speech problems (*n* = 7), and one-sided weakness (*n* = 23). Fifteen patients also experienced nausea, vomiting, and amnesia.

The inclusion criteria were as follows (7–10): (1) a stenosis rate of ≥70% confirmed by computed tomography or magnetic resonance imaging, and stroke-related symptoms; (2) a stenosis rate of ≥50%, and presence of unstable plaques; (3) complete occlusion confirmed by imaging with an occlusion length of ≤10 mm, and stroke-related signs and symptoms; (4) >18 years of age with unilateral stenosis.

The exclusion criteria were as follows: (1) serious systemic disease (e.g., complete loss of brain function on the affected side, paralysis); (2) a carotid artery occlusion of > 10 mm; (3) contraindications to angiography; (4) carotid artery mural thrombosis or multi-segment stenosis; (5) lesions that could not be reached by intraluminal methods (e.g., serious sinuous in the aortic arch branches, absence of a suitable access artery, special aortic arch anatomy); (6) stenosis close to the carotid aneurysm; (7) a history of intracranial hemorrhage in the past 3 months, or a large area of cerebral infarction in the past 4 weeks; (8) moderate or severe stenosis in the bilateral carotid artery.

The study was approved by the ethics committee of the First Affiliated Hospital, and College of Clinical Medicine of Henan University of Science and Technology. Written informed consent was obtained from all participants.

### Ultrasound Examination

Patients underwent carotid artery ultrasound 3 days before ESP. An iE33 color Doppler ultrasound machine and L11-3 vascular probe (Philips, Amsterdam, Netherlands) were used. Patients were instructed to assume a supine position, and the tested side of the neck was exposed. Morphological indices were measured and calculated in the carotid artery by ultrasound. Measurements included the number of unstable plaques (NUP; i.e., number of vulnerable plaques with a large lipid-rich necrotic core, intra-plaque hemorrhage, fibrous caps on the affected side); the unstable plaque index (UPI; i.e., the volume ratio of unstable plaques to stable plaques), the peak eccentricity ratio (PER; i.e., the ratio of the maximum to the minimum artery wall thickness), and the carotid area narrowing rate (ANR; i.e., the cross-sectional area of a blood vessel region with plaque buildup divided by the cross-sectional area of a blood vessel region without plaque buildup).

In this study, lipid plaques with low echo, mixed plaques with obvious uneven echo, ulcerative plaques with uneven surface and bleeding plaques with no echo inside were called unstable plaques, also known as vulnerable plaques.

Hemodynamic indices included the peak systolic velocity (PSV) at the carotid stenosis, the resistance index (RI), and the pulsatility index (PI).

Physiological indices (11–13) included the carotid artery stiffness index (β) on the affected side, the dilation coefficient (DC), and the compliance coefficient (CC).

All examiners were experienced clinicians, and the inter-rater reliability of the variables was 0.75–0.8.

### Evaluation of Neurological Status

The clinical effects of ESP were represented by the neurological deficit IR before and after surgery. The National Institute of Health Stroke Scale (NIHSS) score was used to assess neurological deficit 1 day before ESP and 30 days after ESP. The actual IR was calculated according to the NIHSS score before and after surgery according to the following equation: Actual IR = (NIHSS_before treatment_ – NIHSS_after treatment_) ÷ NIHSS_before treatment_ × 100%. An actual IR of ≥75% was defined as an excellent outcome, an IR of 25–74% was defined as an effective outcome, and an IR of <25% was defined as an ineffective outcome (14, 15).

### Data Processing

SPSS 18.0 was used for K-S test of normal distribution of ultrasonic data, and to analyze correlation between ultrasound indices and the neurological deficit IR after ESP. Pearson correlation analysis was used for normal distribution data and Spearman correlation analysis was used for non-normal distribution. A multiple linear regression equation of the measured ultrasound indices and neurological deficit fitted IR were established. The intergroup comparison of measurement data (mean ± standard deviation [SD] or median [IQR]) and hypothesis testing of the regression equation were conducted using an analysis of variance. The regression coefficient was tested using a *t-*test. The inspection level (α) was 0.05.

## Results

### Clinical Evaluation of the Neurological Status of Patients 30 Days After ESP

Before ESP, 7 out of 64 patients had minor stroke (NIHSS score, <5), 29 out of 64 patients had moderate stroke (5 ≤ NIHSS <15), 17 out of 64 patients had moderate-to-severe stroke (15 ≤ NIHSS <20), and 11 out of 64 patients had severe stroke (NIHSS score, ≥20). The mean ± SD and median (IQR) preoperative NIHSS scores were 19.5 ± 6.4 and 14.5 (8.5), respectively. NIHSS scores were calculated again 30 days after ESP. There was no significant difference in clinical basic data of 64 patients, *P* > 0.05, as shown in Table 1.

**Table 1:** Clinical basic data of 64 patients.

	**N**	**Mean**	**Standard Deviation**	***F*-value**	***P*-value**	
Gender	M = 1	35	1.45	0.50	0.76	0.47
	F = 2	29				
Age		64	65.19	8.23	2.82	0.07
BP		64	138.50	12.86	2.02	0.14
BMI		64	23.87	1.92	0.59	0.56
FBG		64	5.52	0.89	1.75	0.18
LDL-C		64	3.27	0.51	2.20	0.12
Fibrinogen		64	3.05	0.59	0.61	0.54
CRP		64	5.67	1.71	1.62	0.21

According to any observed improvement in neurological deficit after surgery, patients were divided into three groups: excellent (*n* = 23), effective (*n* = 35), and ineffective (*n* = 6). The total percentage of patients in the effective group was 90.6%.

After surgery, the mean ± SD and median (IQR) NIHSS scores were 6.1 ± 2.3 and 6 (4.5), respectively. The mean NIHSS score IR after surgery was 71.25% ± 19.48%. All patients survived the follow-up period (30 days). Two patients experienced recurrent stroke within 3 days of surgery and presented with more severe neurological deficit and carotid artery narrowing. One patient recovered 1 week after surgery and the other showed left-sided weakness, speech problems, and blurred vision during the follow-up period.

### Pearson's Correlation Between Ultrasound Indices and the Neurological Deficit IR

The results of K-S test show that the skewness coefficient and kurtosis coefficient of each ultrasonic index dates are <1, which can be regarded as approximate normal distribution. Of all the measured ultrasound indices, PSV strongly correlated with the actual postoperative IR. UPI, PER, and β moderately correlated with actual IR, while RI and DC weakly correlated with actual IR. Furthermore, Pearson's correlation coefficient showed no statistical difference between NUP, ANR, PI, and CC and the actual IR (*P* > 0.05; Table 2).

**Table 2:** Pearson's correlation between ultrasound index and Actual IR after operation.

**Ultrasound index**	**Mean**	**Standard deviation**	**Pearson's *r***	***P*-value**
NUP	3.92	0.74	0.295	0.167
UPI	2.197	0.952	0.447	0.001
PER	3.520	1.347	0.431	0.001
ANR	71.5	8.325	0.126	0.364
PV	2.707	0.753	0.622	0.000
RI	0.695	0.093	0.325	0.046
PI	2.588	0.889	0.033	0.813
β	3.129	0.996	0.512	0.000
Dc	38.63	15.17	0.311	0.022
Cc	0.4582	0.1415	0.359	0.251

### Ultrasound Prediction of ESP Outcomes

#### The Degree of Confidence of Ultrasound Indices in Predicting the Postoperative Outcomes of ESP

According to different neurological deficit IRs, the postoperative effects of ESP were divided into three categories: excellent, effective, and ineffective. The degree of confidence of ultrasound morphological indices (i.e., NUP, UPI, PER, and ANR) to predict the probability of excellent, effective, and ineffective outcomes after ESP was 11.9, 12.5, and 23.7%, respectively.

The degree of confidence of hemodynamic parameters (i.e., PSV, RI, and PI) to predict the probability of excellent and effective outcomes after ESP was 3.95% and 4.53%, respectively, while the degree of confidence to predict the probability of ineffective outcomes was 39.1%.

The degree of confidence of physiological ultrasound indices (i.e., DC and CC) to predict the probability of excellent outcomes after ESP was 26.7%, while the degree of confidence to predict the probability of effective outcomes after ESP was 4.1%. β could determine ineffective outcomes after ESP. That is, a β value of >4.4759 suggests an ineffective outcome after ESP.

#### Ultrasound Indices to Predict Neurological Deficit IR After ESP

**Figure 1:**
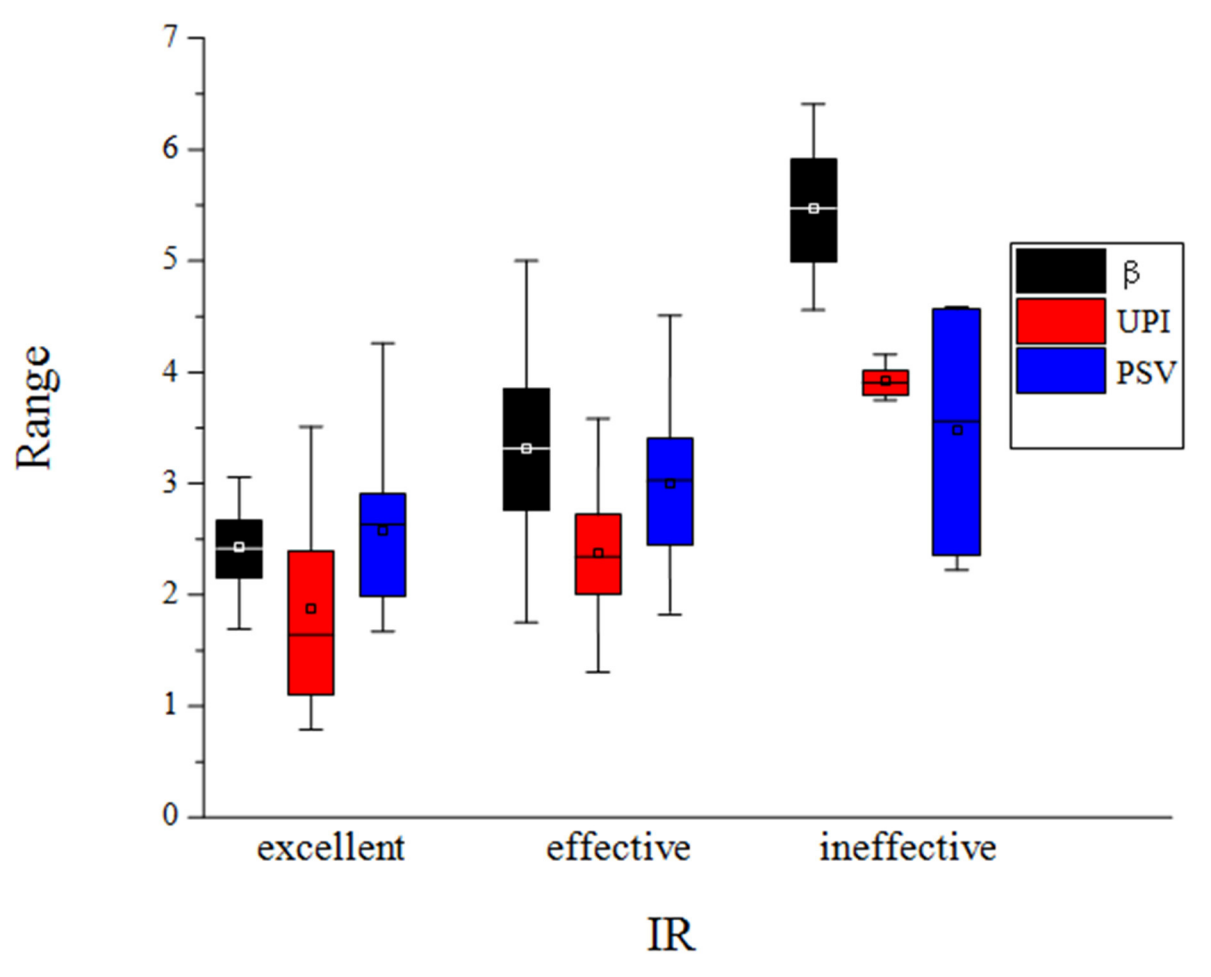
The box diagram of β, UPI and PSV in different treatment effect groups, in which the black box diagram represents the β value, the red box diagram represents the UPI value, and the blue box diagram represents the PSV value.

The predictive ultrasound model using a combination of morphological, hemodynamic, and physiological indices is such that: fitted IR = 0.9816 – 0.1293β + 0.0504UPI – 0.1137PSV (*R*^2^ = 0.6239). Analysis of variance: *F* = 12.41 > F_(3, 60)_ = 4.13 (*P* < 0.01). Therefore, there was a linear correlation between the three indices of preoperative carotid artery ultrasound (β, UPI, and PSV) and the neurological deficit IR after ESP. Hypothesis testing of regression coefficients for each ultrasound index is depicted in Table 3. The box diagram of β, UPI, and PSV in different treatment effect groups is shown in Figure 1.

**Table 3:** Partial regression coefficient *T* test for prediction model of ultrasonic index.

**Variable**	**Parameter estimation**	**Standard error**	***t*-value**	***P*-value**
Intercept	0.9475	0.1428	6.63	<0.01
β	−0.1049	0.0303	−3.56	<0.05
UPI	0.0008	0.0003	2.71	<0.01
PV	−0.0713	0.0408	−1.75	<0.05

### Actual Neurological Deficit IR After ESP in 38 Patients and the Fitted Curve of the Ultrasound Prediction Model

We retrospectively analyzed a new set of patients (*n* = 38) using the predictive ultrasound model before and 30 days after ESP. The median (IQR) preoperative and postoperative NIHSS scores were 17.5 (9.25) and 9 (4.75), respectively. The actual IR was 48.40 ± 11.7%. Prior to surgery, UPI, PV, and β were 2.574 ± 0.534, 2.651 ± 0.667, and 2.579 ± 0.667, respectively, and the predicted IR was 47.6 ± 0.12.8%. The actual IR and the predicted IR significantly correlated with an *R*^2^ coefficient of 0.829 (*P* = 0.006).

### Ultrasound Before and After ESP in Patients With Ideal Fitted Effects Predicted by Ultrasound

**Figure 2:**
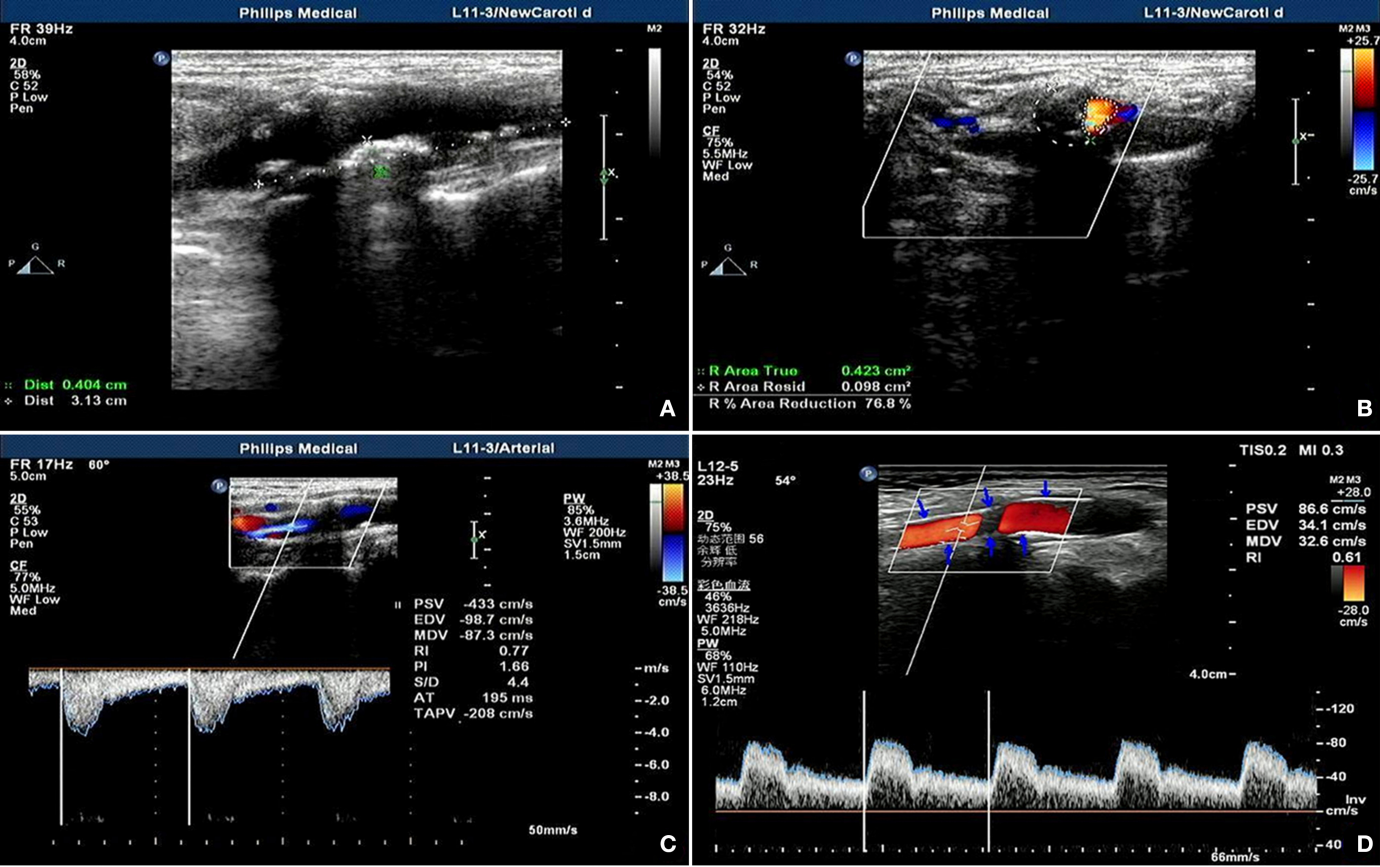
Ultrasonic manifestation before and after coronary artery stenting in patients predicted as having ideal outcomes. **(A)** shows multiple mixed echo mural plaques, some of which are unstable plaques; **(B)** shows cross section of stenosis, showing area stenosis rate of 76.8%; **(C,D)** show stenosis site before and after ESP respectively On the 30th day, the blood flow at the stent site showed that the shape and position of the carotid stent were good (the arrow showed the carotid stent), the blood flow in the stent was smooth, and the PV and RI were decreased.

A 63-year-old male had a preoperative NIHSS score of 13. The patient's NIHSS score 30 days after surgery was two and the patient's actual IR was 84.6%. The ultrasound model predicted a fitted IR of 87.1%. Figure 2A presents a section of the long axis of the vessel before surgery. The image reveals multiple mixed echoes in mural plaques and some unstable plaques. Figure 2B presents a transverse section of the stenosis, revealing an ANR of 76.8%. Figures 2C,D present the blood flow at the stenosed site before ESP and at the stent site 30 days after ESP, respectively. Observations revealed that the stent morphology and position in the carotid artery were good after surgery (the arrowhead indicates the carotid artery stent). In the stent, blood flow was smooth, and PSV and RI were reduced.

### Ultrasound to Predict the Effect of Non-ideal Patients Before and After ESP

**Figure 3:**
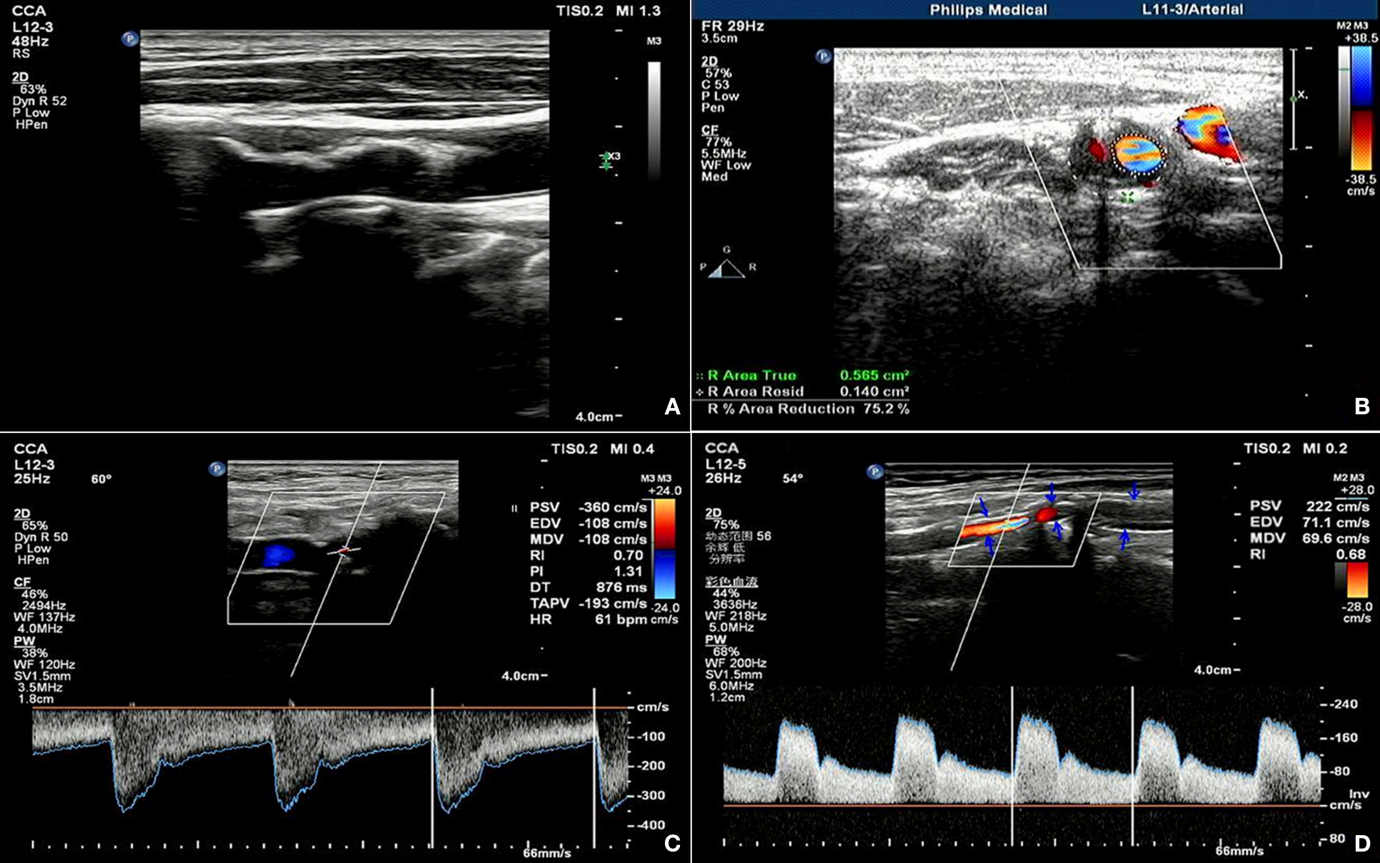
Ultrasonic manifestation before and after coronary artery stenting in patients predicted as having non-ideal outcomes. **(A)** shows the long axis view of the vessel before the operation, showing the strong echo mural plaques in the stenosis; **(B)** shows the transverse view of the stenosis, showing the area stenosis rate of 75.2%; **(C,D)** show the blood flow in the stenosis before the ESP and 30 days after the operation, respectively, showing the poor extension of the carotid stent after the operation, and the blood flow PV and RI in the stent are lower but still higher than before the operation.

A 74-year-old female had a preoperative NIHSS score of 35. The patient's NIHSS score 30 days after surgery was 22. The actual IR was 37.1% and the fitted IR was 48.8%. Figure 3A presents a section of the long axis of the vessel before surgery, which reveals strong echo in mural plaques at the stenosed site. Figure 3B presents a transverse section of the stenosis and reveals an ANR of 75.2%. Figures 3C,D present blood flow at the stenosed site before ESP and at the stent site 30 days after ESP, respectively. These observations reveal that partial carotid artery stents were poorly extended after surgery, and that the PSV and RI of in-stent blood flow decreased when compared with the respective values before surgery, although the values remained relatively high.

## Discussion

The Report on the Prevention and Treatment of Stroke in China (2016) revealed that the prevalence of stroke in China increased from 0.4% in 1993 to 1.23% in 2013. The prevalence has continued to increase in recent years and stroke has become a critical disease that seriously impedes socio-economic development due to its high incidence, high recurrence rate, and high disability rate (16, 17).

Carotid atheromatous plaque formation and atherosclerotic stenosis directly correlate with ischemic stroke. ESP and carotid endarterectomy can decrease the risk of recurrence and progression of ischemic stroke.

Due to its simple surgical approach and minimal surgical trauma, ESP is becoming an increasingly popular surgical option for clinicians in the treatment of stenosis in China, when compared with endarterectomy. However, the value of ESP in the treatment of carotid artery stenosis is not only based on immediate results, but also on the incidence of complications and its effect in the later periods. Methods to predict the effect of ESP before surgery play a positive role in clinical guidance and in selecting optimal treatments.

Magnetic resonance imaging can display calcification of carotid plaques, plaque bleeding, and plaque thrombosis, and can distinguish the core of lipid necrosis from plaques. Thus, magnetic resonance imaging has been recommended as the first choice of examination for coronary artery stenosis before ESP (18, 19). However, magnetic resonance imaging is limited by its imaging principles, and inevitably has a sidedness in the analysis of diseased vessels, except for two-dimensional morphology. Furthermore, magnetic resonance imaging cannot be used to evaluate hemodynamic changes or changes in the physiological elasticity of blood vessels.

On the other hand, ultrasound can reliably display carotid artery wall sclerosis and luminal stenosis and can be used to observe the morphology and properties of atherosclerotic plaques; dynamically detect changes in hemodynamic parameters at carotid artery stenosis sites in real time; and analyze carotid artery stiffness, compliance, and dilatation. Ultrasound can also be used to analyze other physiological indicators, such as arterial blood pressure. Hence, ultrasound can more objectively and comprehensively evaluate blood vessels when compared with other imaging methods. Therefore, ultrasound can be used to predict the outcomes of carotid artery stenosis after stent placement. In particular, intravascular ultrasound, which was developed in recent years, has advanced the diagnosis of vascular disease (20, 21).

The present study revealed that two-dimensional morphological indices of ultrasound (i.e., UPI and PER) moderately correlate with the NIHSS score IR after surgery. Second, in terms of hemodynamic indices, PSV at the stenosed site strongly correlated with the NIHSS score IR, while RI weakly correlated with the NIHSS score IR. Third, in terms of physiological indices, β moderately correlated with the NIHSS score IR, while DC weakly correlated with the NIHSS score IR. These results suggest that the contribution of different indicators to the effect of ESP varies, and that these indicators should occupy different weights in the ultrasonic evaluation of carotid artery lesions. However, NUP, ANR, PI, and CC failed to exceed the inspection level of 0.05. That is, the impact of these four indices on postoperative outcomes remains unclear and requires further verification using larger sample sizes.

According to the NIHSS score IR 30 days after ESP and 1 day before ESP, the effects of ESP on patients 30 days after surgery were divided into three categories: excellent, effective, and ineffective.

The degree of confidence of the morphological index calculated using the statistical formula was 23.7% in predicting the probability of ineffective outcomes after ESP. However, it was difficult to predict the probability of effective and excellent outcomes, and the degree of confidence in predicting these outcomes was 11.9 and 12.5%, respectively.

The hemodynamic index had a higher degree of confidence compared with the morphological index in predicting the probability of ineffective outcomes after ESP (39.1%). Hence, it was also difficult to predict the probability of effective and excellent outcomes using the hemodynamic index, and the degree of confidence of each of these outcomes was 3.95 and 4.53%, respectively.

The physiological index had the highest degree of confidence in predicting the probability of ineffective outcomes after ESP (it could almost determine ineffective outcomes after ESP). That is, when β was >4.4759, all patients achieved ineffective outcomes after ESP, with the exception of one patient. The degree of confidence in predicting the probability of excellent outcomes was 26.7%, which is similar to the ability of morphological and hemodynamic indices to predict the probability of effective outcomes, and the degree of confidence was only 4.1%.

These observations suggests that, compared with the morphological index, hemodynamic and physiological indices have slightly higher degrees of confidence in predicting the effect of ESP. Therefore, when using ultrasound to evaluate the effect of ESP, the morphological index cannot be used unilaterally, and hemodynamic and physiological indices must be combined for comprehensive assessment. Some scholars have also recommended that contrast-enhanced ultrasound should be used to observe more accurate hemodynamic changes in carotid artery stenosis, which would improve the reliability of ultrasound in predicting re-stenosis after ESP (22–24).

In the present study, the combined application of three ultrasound indices improved the degree of confidence in predicting the probability of excellent and ineffective outcomes. However, limited by the small sample size, this approach could not determine the probability of effective outcomes (actual IR, 25–74%) after ESP.

The multiple linear regression model was established using preoperative ultrasound indices, and the neurological deficit IR after ESP revealed a linear correlation between the actual IR and three regression variables: β, UPI, and PSV. The coefficient of determination, *R*^2^, was 0.6239. Therefore, 62.39% of the IR after ESP could be explained by the linear combination of these three preoperative ultrasound indices.

**Figure 4:**
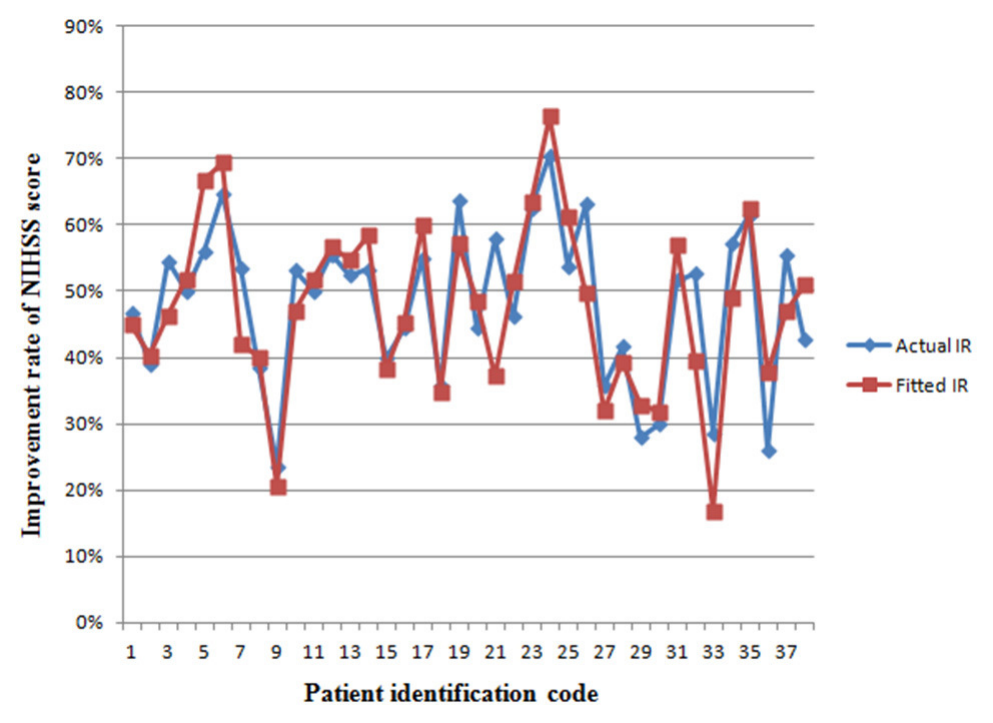
The fitting curve of the improvement rate of NIHSS score in a new set of patients from retrospective sub-study. The red line represents the actual IR of neurological deficit. The blue line represents the fitted values of the model. IR, improvement rate.

Figure 4 reveals that the fitted IR of the model is in line with the distribution of the actual IR curve. This suggests that the preoperative ultrasound examination of these three comprehensive indices would be helpful for the rapid assessment of ESP outcomes. Studies with larger sample sizes may improve the degree of confidence of preoperative ultrasound indices to predict the effects of ESP, and further improve *R*^2^ of the predictive model; that is, the accuracy of prediction.

### Limitations

Due to the small number of samples in this study, especially in the ineffective group, only six patients were collected, which greatly limits the external validity of the associations found between ultrasound indicators and the patient group. We look forward to a larger sample study to further confirm the conclusions of this study.

## Data Availability Statement

The raw data supporting the conclusions of this article will be made available by the authors, without undue reservation.

## Ethics Statement

The studies involving human participants were reviewed and approved by the ethics committee of the First Affiliated Hospital, and College of Clinical Medicine of Henan University of Science and Technology. The patients/participants provided their written informed consent to participate in this study.

## Author Contributions

S-JC: conceptualization, investigation, project administration, supervision, writing - original draft, and writing - review & editing. Y-RS: conceptualization, investigation, and methodology. Y-JX, X-FY, J-SQ, D-YS, and J-YL: investigation and methodology. R-RL: investigation, writing - original draft, and methodology. X-HG, M-JH, and Q-ZF: writing - review & editing. All authors contributed to the article and approved the submitted version.

## Conflict of Interest

The authors declare that the research was conducted in the absence of any commercial or financial relationships that could be construed as a potential conflict of interest.
